# Attitude and knowledge about foot health: a spanish view

**DOI:** 10.1590/1518-8345.1643.2855

**Published:** 2017-04-06

**Authors:** Daniel López-López, Ricardo García-Mira, Patricia Palomo-López, Rubén Sánchez-Gómez, José Ramos-Galván, Natalia Tovaruela-Carrión, Matilde García-Sánchez

**Affiliations:** 1 PhD, Assistant Professor, Facultade de Enfarmaría e Podoloxía, Universidade da Coruña, Ferrol, Spain.; 2 PhD, Full Professor, Facultad de Ciencias da Educación, Universidade da Coruña, A Coruña, Spain.; 3 PhD, Assistant Professor, Centro Universitario de Plasencia, Universidad de Extermadura, Plasencia, Spain.; 4 PhD, Assistant Professor, Facultad de Ciencias de la Salud, Universidad Europea de Madrid, Madrid, Spain.; 5 PhD, Full Professor, Facultad de Enfermería Fisioterapia y Podología, Universidad de Sevilla, Sevilla, Spain.; 6 PhD, Assistant Professor, Facultad de Enfermería Fisioterapia y Podología, Universidad de Sevilla, Sevilla, Spain.; 7 PhD, Full Professor, Facultade de Enfarmaría e Podoloxía, Universidade da Coruña, Ferrol, Spain.

**Keywords:** Foot, Perception, Podiatry

## Abstract

**Objective:**

to explore attitudes towards patients' self-reported data about foot health-related beliefs from a behavioural and attitudinal perspective.

**Methods:**

a sample of 282 participants of a mean age of 39.46 ± 16.026 came to a health centre where self-reported demographic, clinical characteristics and beliefs relating to foot health data were registered, informants' completed all the stages of the research process.

**Results:**

the results of the analysis revealed an 8-factor factorial structure based on (1) podiatric behaviours, (2) the intention to carry out protective behaviour, (3) attitudinal beliefs, (4) normative beliefs, (5) needs, (6) apathy, (7) self-care, and (8) the general perception of foot health. They all explained 62.78% of the variance, and were considered as independent variables in a regression analysis to determine which provided the best explanations for the importance attributed to foot health.

**Conclusions:**

the participants in the study revealed a positive attitude in relation to foot health care and responsible behaviour.

## Introduction

The increase in life expectancy and the high prevalence of foot pathologies related to obesity, diabetes, the practice of sport, vascular alterations, physical injury and a sedentary lifestyle^1^ for which there is no total cure and where the therapeutic goal is to relieve or eliminate symptoms, avoid complications and improve the patient's wellbeing, means that classical medical measurements of outcome (mortality, morbidity, life expectancy) are insufficient to provide a thorough assessment of whether patients receive appropriate and effective treatment for foot diseases.

Also, such problems currently affect between 71 and 93% of general population and are a frequent cause of medical and foot care^2^ since they have been shown to be neither minor nor banal and have a negative influence on functional capacity and quality of life^3^ ^-^ ^5^. These conditions are multifactorial in their origin and their high incidence was related with difficulty in putting shoes, pain, gait disturbance, reduced walking speed, variation in plantar pressures, risk of falls^6^ ^-^ ^8^. The pathologies and alterations more frequently found were claw toes, hallux valgus, hammer toes, overlapping toes, hallux extensus, pes planus, morton's neuroma, tailor's bunions, plantar fascitis and pes cavus^2^ ^,^ ^9^.

The research questions addressed therefore concern the following aspects: what attitudes and factors influence people's perception of foot diseases and the health professional who treat them? What are the most suitable methods we can use to increase our knowledge of these attitudinal aspects?

In order to attempt to provide an answer to these questions the overall research objective was defined as being to evaluate the social representations of foot health and the podiatric and psychological aspects involved in the analysis of human behavior.

We will thus be able to perceive whether the main motive is related to the negative impact of foot diseases on functional capacity and quality of life^10^ and in this regard the main tool for the analysis of health research is the construction of questionnaires on a scientific basis^11^ as a reliable method of measuring results and generating clinical evidence, 

The importance of a study of this kind lies in the possibility of analyzing particular behaviors and our knowledge of the psychosocial context, since they can potentially generate a risk of suffering from foot pathologies.

This will have a positive influence on patients' response and adherence to treatment characterized by the introduction of a variety of activities that people carry out in their everyday lives and the importance attributed to disease in general, which people will also apply to its causes^12^. All of this has an effect on the type of preventive behaviors that accompany a treatment or lessen the possibility of being affected by a foot or ankle pathology^13^.

In this regard, the present study analyzes beliefs relating to foot health, from a behavioral and attitudinal standpoint, due to the lack of knowledge of the criteria people take into account when evaluating the seriousness of everything that affects foot health.

## Method

### Design and sample

The overall study was completed in 12 months from January 2014 to January 2015. The study was carried out among people at Clinic of Podiatric Medicine and Surgery that provides treatment of diseases and disorders of the foot at University of A Coruña in the city of Ferrol (Spain).

It was a cross sectional study. A consecutive sampling method was used to select study participants. The inclusion criterion being aged 65 or less and provided informed consent to participate. The exclusion criterion was a history of major, psychiatric disease, dementia, neurological disorders, immunocompromised patients, trauma and a history of foot surgery and refusal to sign the consent form or incapable of understanding the instructions necessary to carry out the present study.

### Procedure

At enrolment, patients were interviewed about general health, demographic characteristics (age, gender, marital status, income, education). A single trained examiner performed a standardized clinical exam on all participants who first measured height, weight with the subject barefoot and wearing light clothing and the body mass index (BMI) was calculated from the height (m) and weight (kg^2)^, applying following Quetelet's equation BMI=weight / height²^14^.

In the second place, to determine the attitudes towards patients' self-reported data about foot health-related beliefs from a behavioral and attitudinal perspective using a *ad hoc* questionnaire was designed in order to collect precise data about the subject's profile in general, together with a series of specific features defining it. Data were also gathered regarding lifestyle-related attitudes and behaviors, everyday habits, the assessment of subjective relevance, preventive behaviors and the social perception of podiatry, these being an important factor in maintaining foot health and of relevance when determining which specific aspects are most closely related to this particular form of wellbeing.

**Figure 1:**
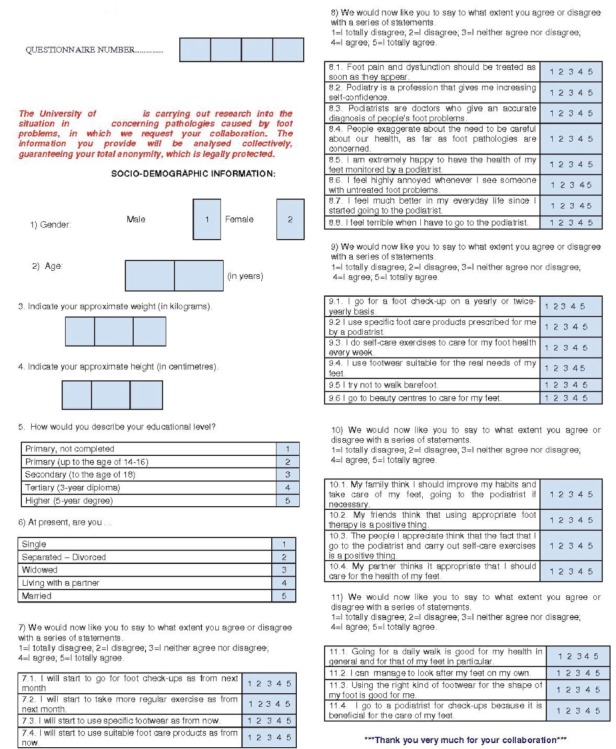
Ad hoc questionnaire. A Coruña, Spain, 2014

A questionnaire was applied that included a set of items that measured the above-mentioned variables on two types of scale: 1) qualitative scales, with open items to collect information on habits and activities; 2) 5-point Likert-type scales, to measure the degree of importance attached by subjects to foot health in general, as well as to podiatrists and their situation within the health care system in particular are shown in Figure 1.

This research was reviewed by the Research and Ethics Committee of the University of a Corunna, Spain, which was approved with CE 06/2014 registration number.

## Statistical analysis

### Sample size

The sample size was calculated with the software from Clinical Epidemiology and Biostatistics Unit.or the University of A Corunna^15^. By the sample target size for a bilateral hypothesis, an alpha risk of 5% and a statistical power of 80%, and a beta error of 20%, at least 282 cases must be studied

Descriptive analyses, including calculation of means, standard deviations (SD), and ranges were calculated for quantitative variables: age, weight, height and BMI. A principal component factorial analysis was also performed, in order to obtain a factorial structure that makes it possible to explore and determine the dimensions that characterize people's perceptive model of podiatry and foot health from the point of view of attitudinal, normative, intentional and behavioral beliefs, from a theoretical planned action perspective.

The final stage was to carry out a multiple linear regression analysis, using the Stepwise method, taking the various factors as independent variables and the '*importance attributed to foot health*' as the dependent variable. The purpose of this was to determine which factors contributed most to the assessment of attributed importance, and the tool used was the SPSS package (version 16), for descriptive and statistical analysis, with significance level lower than 5%.

## Results

A total of 282 people completed all the stages of the research process, 80 of them were men (28.4%) and 202 women (71.6%). Their ages ranged from 12 to 90, the mean age being 39.46 ± 16.026 years, 66.28 ± 12.126 in weight, 166.4 ± 7.846 cm in height, BMI = 23.94 ± 4.51 kg/m^2^, who have completed a three-year diploma course, married and currently working.

Factorial analysis generated by the method of principal components with Varimax rotation, based on the 26 items and obtained from the sample revealed the existence of 8 factors that explain 62.8% of the variance (Table 1).

**Table 1:** Matrix of rotated components concerning the perception of foot care. A Coruña, Spain, 2014

	Component							
	Foot care behaviour ( 1)	Behavioural intention (2)	Normative beliefs (3)	Attitudinal beliefs (4)	Real needs (5)	Apathy towards foot care (6)	Self care (7)	Health perception (8)
31.1 Regular foot check-ups.	.831							
31.2 Specific foot care products	.776							
34.4 Benefit from foot check-ups	.735							
30.7 Well-being deriving from foot check-up	.584							
30.5 Satisfaction deriving from foot check-up	.496			.472				
33.4 I am starting to use specific products		.821						
33.3 I am starting to use appropriate footwear		.800						
33.1 I am starting to go for foot check-ups	.419	.743						
33.2 Friends recommend the importance of foot care		.670						
32.3 The people around me think I should look after my feet			.759					
32.2. My friends think that my looking after my feet is a good thing			.710					
32.1 My family think I need to look after my feet			.686					
32.4 My partner thinks I take proper care of my feet			.662					
30.2. Looking after my feet makes me feel self-confident				.761				
30.6 I feel annoyed when I meet people who do not get their foot problems treated				.616				
30.3 Podiatrists are doctors who specialise in feet				.601				
31.5 I try not to walk barefoot				.452				
30.1 Foot pain should be treated promptly								
31.4. I use appropriate footwear for my feet					.790			
34.3. Using appropriate footwear is beneficial					.722			
30.4. People exaggerate when it comes to looking after their feet						.754		
30.8 I feel bad about going to the podiatrist	.407					.563		
31.6 I go to beauty centres to take care of my feet						.555		
34.2 The extent to which I can look after my own feet							.717	
31.3. I do exercises to strengthen my feet	.408						.482	
34.1. Walking is important for general health and the health of your feet								.794

We will now take a more detailed look at the significance of the outcome of this factorial analysis. The criterion used to extract factors was to retain all factors with an eigenvalue higher than 1. The outcome could have been simplified had we increased this value, but we opted for the traditional criterion in order to maintain maximum variance and to obtain a more significant and easier to use set of 8 aspects related to the perception of foot care from the original instrument (Table 2).

**Table 2:** Breakdown of total variance. Extraction method: Principal component analysis. A Coruña, Spain, 2014

Component	Initial eigenvalues		
	Total	% of variance	Cumulative %
(1) Foot care behaviors	6.057	23.297	23.297
(2) Behavioral intention	2.276	8.754	32.051
(3) Normative beliefs	1.832	7.047	39.097
(4) Attitudinal beliefs	1.542	5.930	45.028
(5) Real needs	1.306	5.023	50.050
(6) Apathy towards foot care	1.192	4.586	54.637
(7) Self-care	1.078	4.145	58.782
(8) Health perception related to moving on foot	1.038	3.994	62.776

Bearing in mind the high communality values (i.e. the proportion of variance explained by the factors) we will consider each of the 8 items in turn in the following analyses.

1) Foot care behaviors: the first factor (23.3% of total variance) brings together those items linked to preventive foot care and well-being, both in general and of the foot in particular. Both are of major relevance for the acquisition of knowledge and the development of the necessary confidence and competence for this to take place. We thus refer to this factor as '*foot care behaviors*'.
2) Behavioral intention: the second factor (8.75% of total variance) includes those variables that have to do with people's knowledge and perceptions of foot health, and whether or not they coincide with the characteristics of the disease, which play a key role in the patient's participation in foot self-care.
3) Normative beliefs: the third factor (7.05% of total variance) brings together the items related to the psychosocial context, this generates a positive response to the therapeutic intervention. Hence the need to study the individual context, because the consideration of the disease, and the personal consideration, in general, of its causes influences the type of preventive behaviors that accompany a treatment or reduce the possibility of suffering a foot pathology.
4) Attitudinal beliefs: the fourth factor (5.93% of total variance) reveals how much people know about foot health and the self-imposed limitations on their lifestyle. Patients who think they are healthy hide their real behavior to avoid a negative response from their doctor, thus enabling them to do as they wish. Those patients that follow the established guidelines are satisfied with their health and have a more fluent communication with health professionals.
5) Real needs: The fifth factor (5.02% of total variance) explicitly seeks to make changes in the modification of our behavior and to enhance it. The use of footwear has acquired a protective dimension and makes it easier to move on foot in Western culture, although sometimes poor praxis is directly linked to falls, alterations to gait and the appearance or worsening of foot pathologies.
6) Apathy towards foot care: The sixth factor (4.59% of total variance) represents the importance that people give to foot care in particular, and healthcare in general, acting as an early means of selective diagnosis. Thus, patients who think they are healthy hide their real behavior to avoid a negative response from their doctor, thereby enabling them to do as they wish.
7) Self care: the seventh factor (4.15% of total variance) reveals whether the patient's knowledge and perceptions coincide or not with the disease's characteristics, playing a key role in the patient's participation in looking after his or her own feet.

Furthermore, self-care ensures the acquisition of confidence and enables greater involvement in the risk management of foot health and a quest for changes to individual health-promoting behaviors allowing certain population groups, such as children, diabetics and the elderly, to obtain a greater benefit.

8) Perception of health related to moving around on foot: The eighth factor (3.99% of total variance) is considered in its own right, as the result of various studies that have proved that physical activities that are part of work and recreational activities result in benefits for health, improving or maintaining physical fitness. This factor can thus help to prevent cardiovascular pathologies and contribute to a decrease in mortality.

Analysis of the importance attributed to foot health as determined by multiple regression analysis, provided information regarding the factors that most contributed to this determination of importance by the subjects in the study.

Thus, taking as variables the 8 factors extracted by means of factorial analysis, and as dependent variable the importance attributed to foot care by those interviewed, the following results were obtained (Table 3).

**Table 3:** The importance attributed to foot health. A Coruña, Spain, 2014

Model	R Cambio en R cuadrado	R Square Cambio en F	Adjusted R Square gl1	Std. Error of the Estimate gl2	Change Statistics				
					Sig.F Change	R Square Change	F Change	df1	df2
F1_Foot care behav'rs	.287(a)	.082	.079	.824	.082	25.151	1	280	.000
F4_Attitudinal beliefs	.362(b)	.131	.124	.803	.048	15.505	1	279	.000
F8_Hlth. perc. Walking	.383(c)	.147	.138	.797	.016	5.266	1	278	.022
F5_Real needs	.403(d)	.163	.151	.791	.016	5.223	1	277	.023
F2_Behav'rl intention	.420(e)	.176	.161	.786	.013	4.475	1	276	.035

The factors that contributed to the attribution of importance to foot health were those that entered the regression equation, namely factors 1, 2, 4, 5 and 8, which between them explained 16.1 % of the variance.

**Figure 2:**
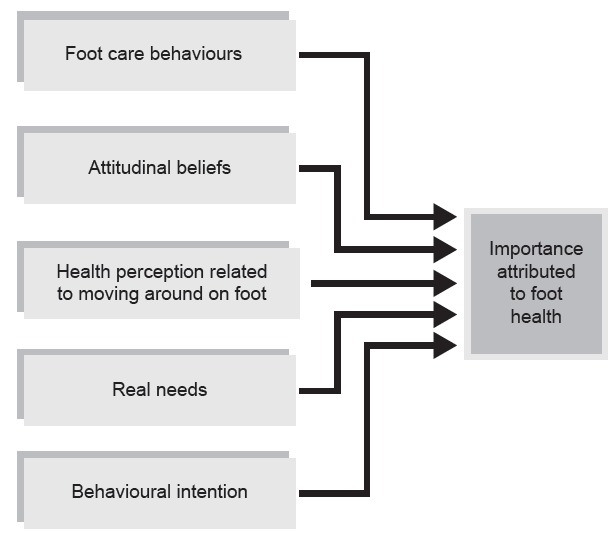
The importance attached to foot health. A Coruña, ES, 2014

Factor 1, "*Foot care behaviors*", contributed to the explanation of the importance attached to foot health with 7.9% of the variance. This was followed by Factor 4, "*Attitudinal beliefs*" (increasing the variance to 12.4%), Factor 8, "*Health perception related to moving around on foot*" (which raised the variance to 13.8%), Factor 5, "*Real needs*" (15.1%), and finally Factor 2, "*Behavioral intention*", which established the variance at 16.1% to explain the importance attributed to foot health (see Figure 2).

The most significant finding derived from these results is that the attribution of importance to foot health is determined by the strength of the following factors: the influence of behavior on subjects' assessment; attitudinal beliefs; the perception of the linkage between moving around on foot and health; and the existence of real needs to visit a health professional that will help to improve health in general, and foot health in particular. These all provide a wealth of information related to preventive or therapeutic behaviors that lead to better health and an improved quality of life.

## Discussion

The response of subjects to disease depends on their previously held image of it, and the persons providing treatments act in a socio-cultural system that legitimizes their behaviors, and also assume a series of socially accepted roles and responsibilities.

In this regard, the response given by subjects to the attribution of the importance of foot health is determined not only by the influence exerted by behavior on the subjects' assessment, but also by that of attitudinal beliefs, health perception associated with moving around on foot, real needs and the intention to carry out self-care behaviors^16^ ^-^ ^18^.

The perception of disease in relation to foot health thus builds confidence and self-assurance that contribute to achieving a healthy lifestyle and avoiding situations of dependency, with moving around on foot being a vitally important habit in order to maintain fitness and prevent both physical and cognitive deterioration^19^ ^-^ ^21^.

In this regard, the attitudinal and normative dimensions play a significant role in the interpretation of human behavior vis-à-vis foot health^22^. The participants in this study reveal the existence of real needs to visit a podiatrist and a demand for foot check-ups by a foot health professional, since it enables people to acquire the confidence and self-assurance needed to maintain their individual foot health and contribute to an improvement in underlying diseases and their general state of health, thereby helping them to lead a healthy life and avoid situations of dependency. Regular check-ups are therefore seen as the preventive behavior that generates the highest degree of self-confidence and with which participants show the greatest agreement, revealing a positive attitude to foot health care and responsible behavior^23^.

This positive attitude is influenced by the increase in life expectancy, the increase in chronic diseases of multifactorial origin and the commitment of podiatry and podiatrists to the management of foot health risk^24^.

We were therefore able to see that there is growing acceptance of podiatry and podiatrists in people's lives and personal activities, integrated into and conceptualized as part of a healthier lifestyle.

## Conclusions

The present study revealed that people's attitudes and beliefs concerning foot health are related to the existence of real needs to visit a podiatrist and the demand for this kind of foot health professional to monitor foot health. This is the result of the existence of a positive social attitude in relation to podiatry and podiatric behaviors that increases the self-assurance and confidence needed to maintain their individual foot health and contribute to an improvement in underlying diseases and their general state of health, thereby helping them to lead a healthy life and avoid situations of dependency.
